# A bibliometric study of identity construction in English writing for academic purposes

**DOI:** 10.3389/fpsyg.2024.1499917

**Published:** 2024-12-10

**Authors:** Yuhan Tian, Donghong Liu

**Affiliations:** School of Foreign Languages, Southeast University, Nanjing, China

**Keywords:** bibliometric study, identity construction, English writing, academic purpose, *CiteSpace*

## Abstract

Identity construction is a crucial factor in assessing and enhancing the quality of academic writing. However, identity is elusive and difficult to capture due to its abstract nature. Most existing literature discussed academic writing in a general way, overlooking specific studies on identity construction in articles, theses, and dissertations. This study aims to provide a comprehensive review of studies on identity construction in typical academic writing and assist readers in understanding the development, discoveries, and future trends in this field. It seeks to enlighten scholars and students regarding future research directions and to improve academic writing quality in practice. A bibliometric tool, *CiteSpace*, was used together with manual close reading. The data were primarily retrieved from the Web of Science database. Keyword co-occurrence and cluster analyses were conducted to describe the current state of research and predict future hotspots. It was found that the literature in this field generally showed an upward trend before 2020. High-frequency keywords primarily relate to literacy, doctoral education, pedagogy, plagiarism, and gender, representing this field’s primary research area. Most clusters exhibit a high level of novelty but have not yet received the attention they deserve because they are situated in the second quadrant of the coordinate diagram as potential clusters. Clusters focusing on socio-cultural identity and the pedagogy of identity construction are more prominent than the other areas. Those focusing on academic (professional) development related to authorial and academic identity are more novel.

## 1 Introduction

Identity has long been a prominent topic in academic writing within higher education. Ivanič (1998) categorized the various textual identities of a writer that require integration, including the autobiographical self, discoursal self, self as author, and possibilities for self-hood. More specifically, identity can be categorized into three dimensions: authorial identity constructed in text, academic (professional) identity developed in higher education, and socio-cultural identity reflected in the writing. The authorial identity is defined as “the sense a writer has of themselves as an author and the textual identity they construct in their writing” (Pittam et al., 2009, p. 154). Academic (professional) identity refers to the researchers’ recognition of themselves as a part of the academic community (Botelho de Magalhães et al., 2019). Authors’ socio-cultural identities encompass their authentic individual identities within society, such as gender, class, and ethnicity, which inevitably influence and are reflected in their academic identity and writing.

Authorial identity contributes to a credible and professional image for authors, thereby enhancing the persuasiveness and quality of their articles. Significant progress has been made in studies on authorial identity, primarily as a pedagogical approach to help students improve academic writing in higher education. It has been demonstrated that enhancing students’ authorial identity could help prevent plagiarism in academic writing (Khoo and Kang, 2022). Various measures of authorial identity have been discussed to ensure the effective prevention of plagiarism and shed light on the practical implications of authorial identity construction (Cheung et al., 2017; Ballantine et al., 2018).

Academic (professional) identity determines the extent to which writers are willing to conform to the norms and conventions of academic writing (Botelho de Magalhães et al., 2019). Studies on academic identity primarily adopt a scholarly approach, focusing on the academic development of scholars in higher education. Academic identity is continuously constructed and reconstructed across various higher education institutions as part of professional development (French, 2020). This spatial dynamics of academic identity is shaped by the spatial nature of academic writing practices (Beighton, 2020), embodied in writing groups and retreats. These practices greatly benefit participants’ academic development and well-being, involving doctoral students, academic returnees, novice lecturers, and experienced teachers.

A comprehensive and nuanced understanding of socio-cultural identities leads to authors’ more mature and flexible use of the language reservoir in their academic writing. Studies on authors’ socio-cultural identities center on the issue of how aspects such as language, gender, class, and ethnicity are reflected in and influence their academic identity and writing. The language aspect in the context of multilingualism is particularly significant due to the globalization of the academic community. Authors’ multilingual skills should be seen as valuable resources rather than challenges to overcome in the process of constructing their identities, which can effectively prevent feelings of inferiority and inequality (Liu and Tannacito, 2013).

The solid construction and skillful integration of identities across all three dimensions mentioned above in academic writing contribute to producing high-quality articles and theses. Writing enables greater deliberation and precision than speaking, allowing identity to be effectively embodied and captured. It serves as the primary means of expressing viewpoints and engaging with audiences in academic contexts. Identity construction involves both the approach and the purpose of enhancing the quality and persuasiveness of academic writing.

Identity construction is a significant factor influencing reviewers’ evaluations of writing quality. High-quality academic output is crucial for scholars’ professional development and students’ attainment of degrees in higher education. Higher education institutions require scholars to publish high-quality articles in reputable journals. Similarly, students must continuously improve their academic writing and complete a demanding thesis or dissertation to attain their degrees.

Raitskaya and Tikhonova (2022) have pointed out that “identity” together with “teaching and learning academic writing in higher education,” “writing for publication,” and “writing a thesis” are among the 25 most frequent keywords in their systematic review of academic writing. However, no systematic review has been conducted on the combined field of these four keywords, specifically focusing on identity construction in articles, theses, and dissertations writing in higher education. Academic writing, including publications and theses, is essential for scholars and students in higher education. The effective construction and strategic use of identity significantly enhance the quality of academic writing, thereby fostering their development within the academic environment. And most of the existing literature employed qualitative methods, such as narrative analysis and thematic analysis, to examine data from comparative cases, questionnaires, and interviews, while also using autobiography and duoethnography to reflect individual perspectives and experiences. Therefore, their research findings carry a certain degree of subjectivity. The quantitative characteristics of this bibliometric study enhance its objectivity. Given its practical and theoretical implications, reviewing the combined field is necessary and significant.

In light of this gap, this study will provide a comprehensive summary and analysis of current research findings using the bibliometric tool *CiteSpace*, offering a more objective understanding of the development, discoveries, and future trends in identity construction in English academic writing in higher education. The focus will be on traditional academic writing, including articles, theses, and dissertations, which exhibit the most salient characteristics of scholarly work. Atypical genres, such as reflective writing and autoethnography, will only be deemed approaches to explore typical academic production in the screened articles.

Specifically, this study aims to address the following three questions:

1. What are the stages of development in the studies on identity construction in academic writing, and what is the general trend?
2. What are the focal areas in the studies on identity construction?
3. What are the current hotspots and potential future ones?

The findings of this study contribute not only to a deeper understanding of identity construction in English academic writing but also to the effective enhancement of writing quality and development for scholars and students, holding great theoretical and practical significance.

## 2 Methods

### 2.1 Data sources, search strategy, and data extraction

**Figure 1:**
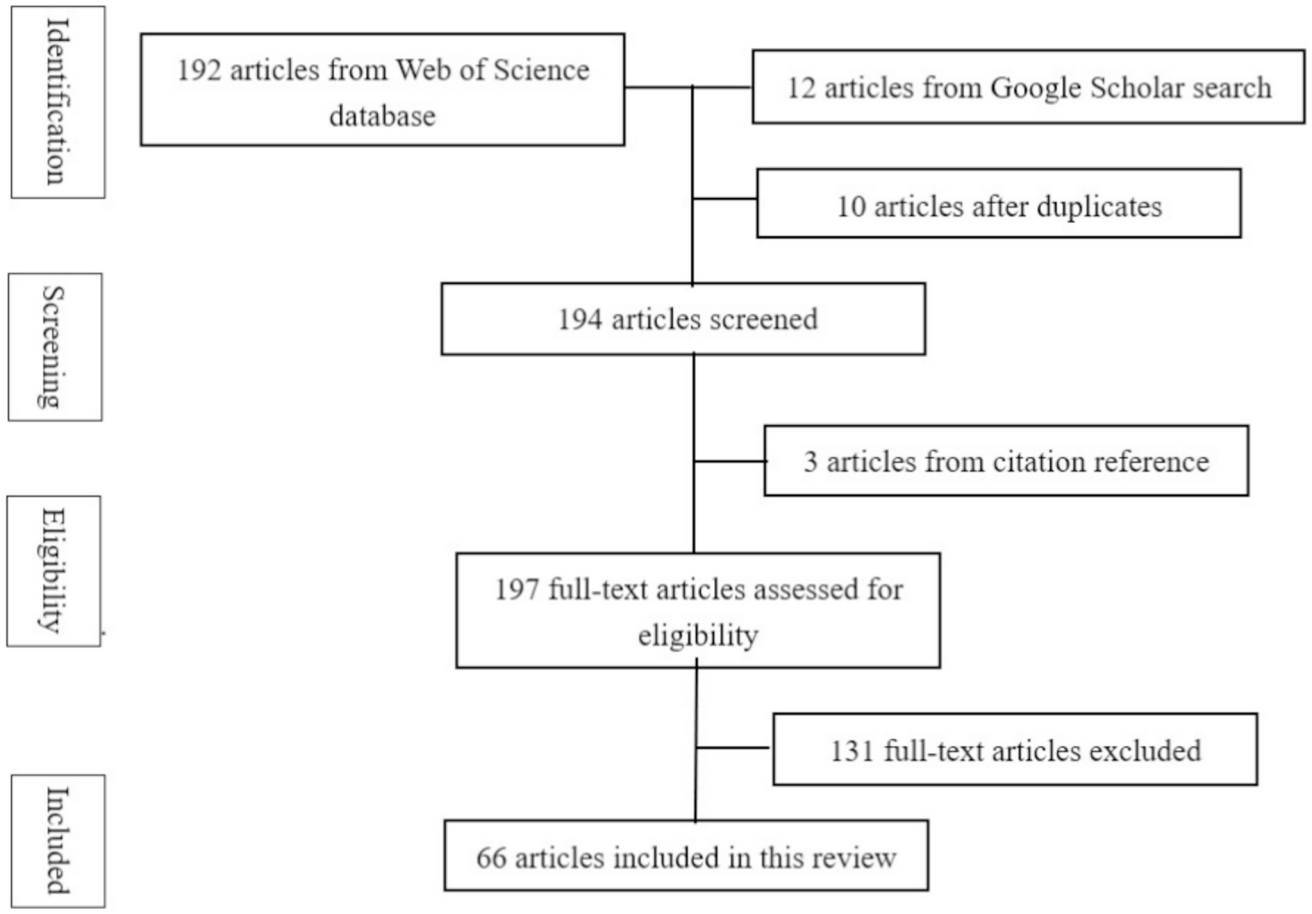
Flow of information through the different phases of systematic review.

First, English-language journal articles from the Core Collections of Web of Science (WoS) database were retrieved as data from *CiteSpace* and close reading. Web of Science is a comprehensive research database renowned for its rigorous indexing and high-quality content. It provides valuable resources for researchers through its advanced search features and extensive coverage. The Core Collection within Web of Science comprises a curated selection of high-quality journals that feature reliable and impactful studies. To ensure the completeness of data and the integrity of the publication year, the time span was set from 1992.1.1, when articles became available for retrieval in this database, to 2023.12.31. The terms “identity,” “academic writing,” and “higher education” were selected to retrieve relevant articles. Additionally, we limited the retrieved data to the type “article” and the language “English” to ensure that only English articles were obtained. Second, citation searches from the references of the above-acquired articles and manual searches from Google Scholar were also employed to supplement any omitted literature. Five more articles have been searched and added to the entire dataset, as shown in Figure 1.

Manual close reading was interspersed throughout the entire study process to screen the targeted literature according to inclusion and exclusion criteria and to generalize conclusions to supplement the results obtained from running *CiteSpace* more elaborately.

To accurately target the articles for review, the retained articles should meet the requirements that (1) studies were related to articles, theses, and dissertation writing; (2) studies were targeted to identity construction; (3) studies were involved in the environment of higher education. Out of 192 articles retrieved from Wos database, 131 irrelevant documents were manually eliminated, owing to their irrelevant foci, including (1) studies without focus on articles, theses, and dissertation writing but on some other atypical academic genres such as reflective writing; (2) studies only focusing on construction of professional identities such as lawyer and tutor or autobiographical identities such as gender, ethnic, and racial identities but failing to relate identity with academic writing; (3) studies without focus on higher education level.

Both authors conducted the screening processes independently. After the respective screenings, we compared the results, identified any inconsistencies, and reached an agreement through negotiation to determine the final articles for analysis. In the end, 61 pieces of data from the WoS database were retained for the final analysis. Five additional articles from citation references and Google searches were included, totaling 66 articles considered in the final analysis. The detailed information for all 66 articles is presented in Supplementary material.

### 2.2 Procedure

First, the data from WoS underwent keyword co-occurrence and cluster analysis by *CiteSpace*. It stands out as a robust bibliometrics tool employed extensively for data analysis and visualization. It can handle bibliographic and citation information sourced from major databases like the Web of Science (Chen, 2006).

A knowledge map will be produced through co-occurrence analysis to illustrate the knowledge structure regarding high-frequency keywords. This will enable us to gain a deeper and more comprehensive understanding of this field’s current state of research.

The relevant literature will be divided into clusters through cluster analysis, revealing their internal structure. These categorized clusters will be plotted on a strategic coordinate graph, with popularity and novelty represented on the horizontal and vertical axes. This will clearly present current research hotspots (clusters in the first quadrant) and predict potential future hotspots (clusters in the second quadrant).

Second, data obtained through citation searches from the references of previously acquired articles and manual searches on Google Scholar underwent close reading to supplement any omissions found in the literature acquired from WoS. Close reading is integrated throughout the entire research process in conjunction with the *CiteSpace* tool.

## 3 Results

### 3.1 Annual distribution

**Figure 2:**
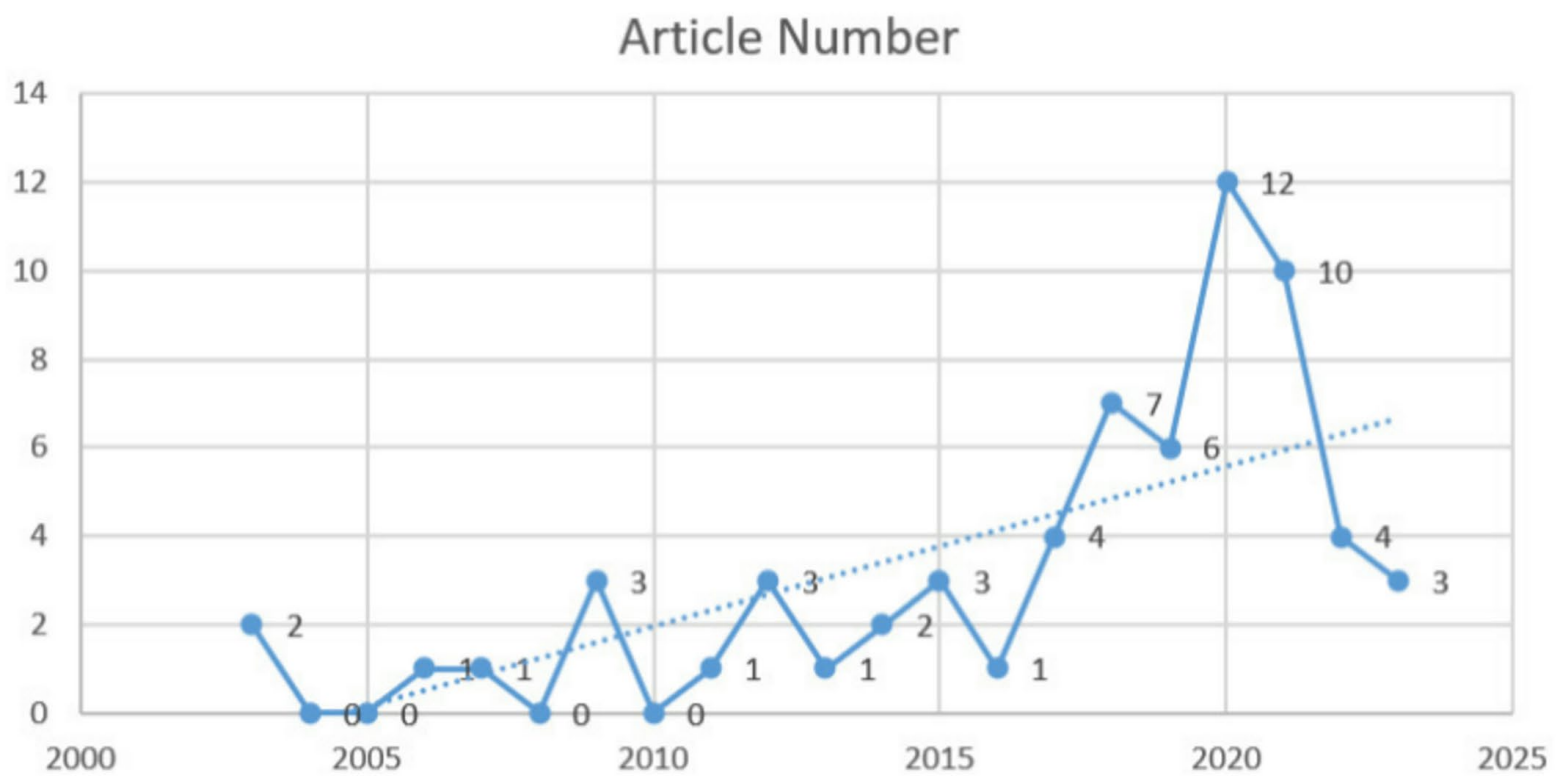
Chronological distribution of publication.

All these articles were distributed over 21 years, as shown in Figure 2, starting from 2003, when the first article was published. It can be concluded that before 2016, articles in this field showed a slight increase amidst fluctuations. The overall data was relatively small, with only 18 articles produced over the 14 years. From 2017 to 2020, the number of articles increased significantly compared to the previous stage. It sharply increased starting in 2017 and peaked in 2020. Since then, it experienced a sharp decline until 2022. From the dotted trend line, it can be seen that the literature in this field generally showed an upward trend before 2020.

### 3.2 Visualization of keyword co-occurrence

**Figure 3:**
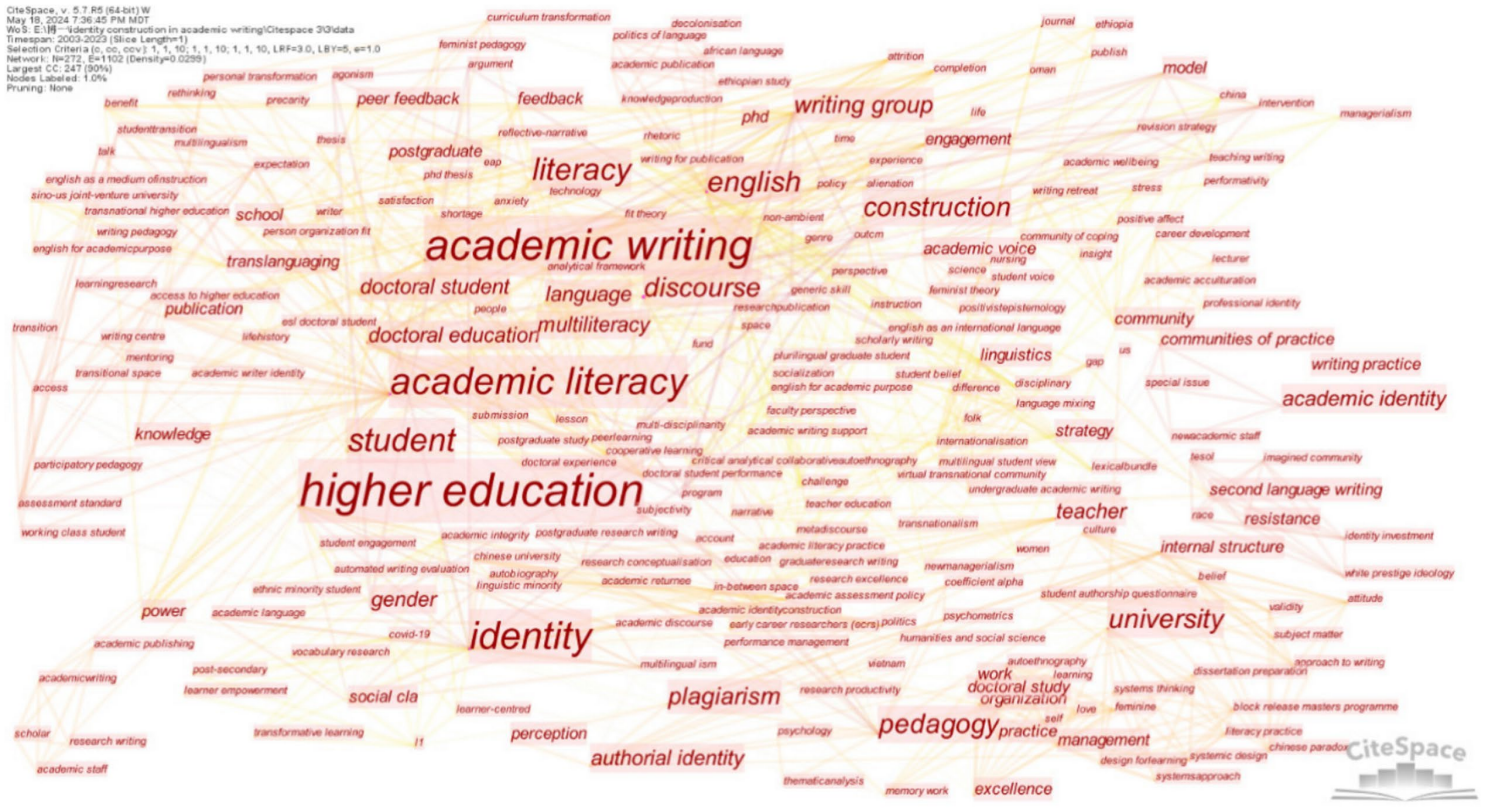
Keyword co-occurrence map.

**Figure 4:**
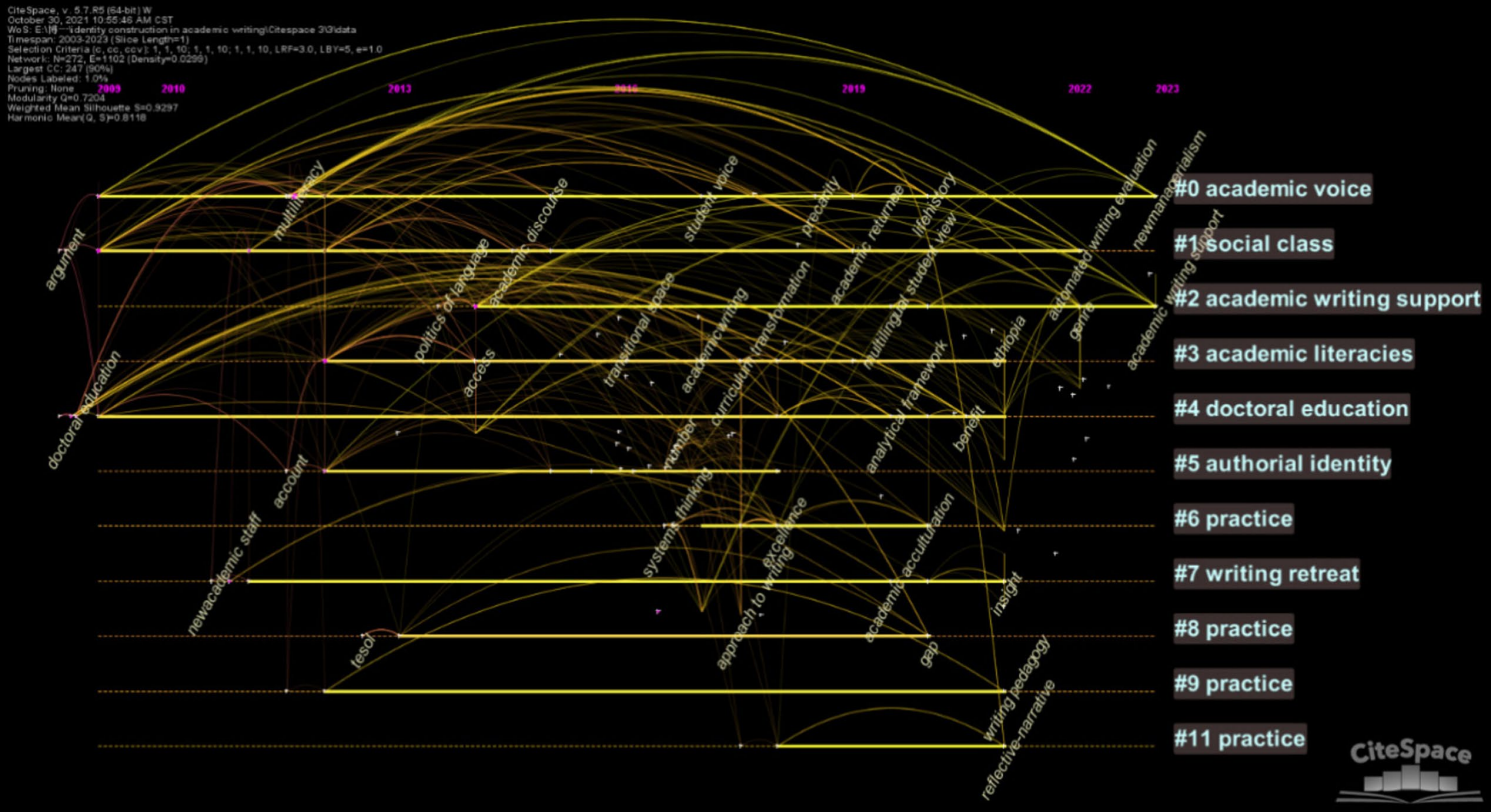
Keywords timeline visualization.

**Figure 5:**
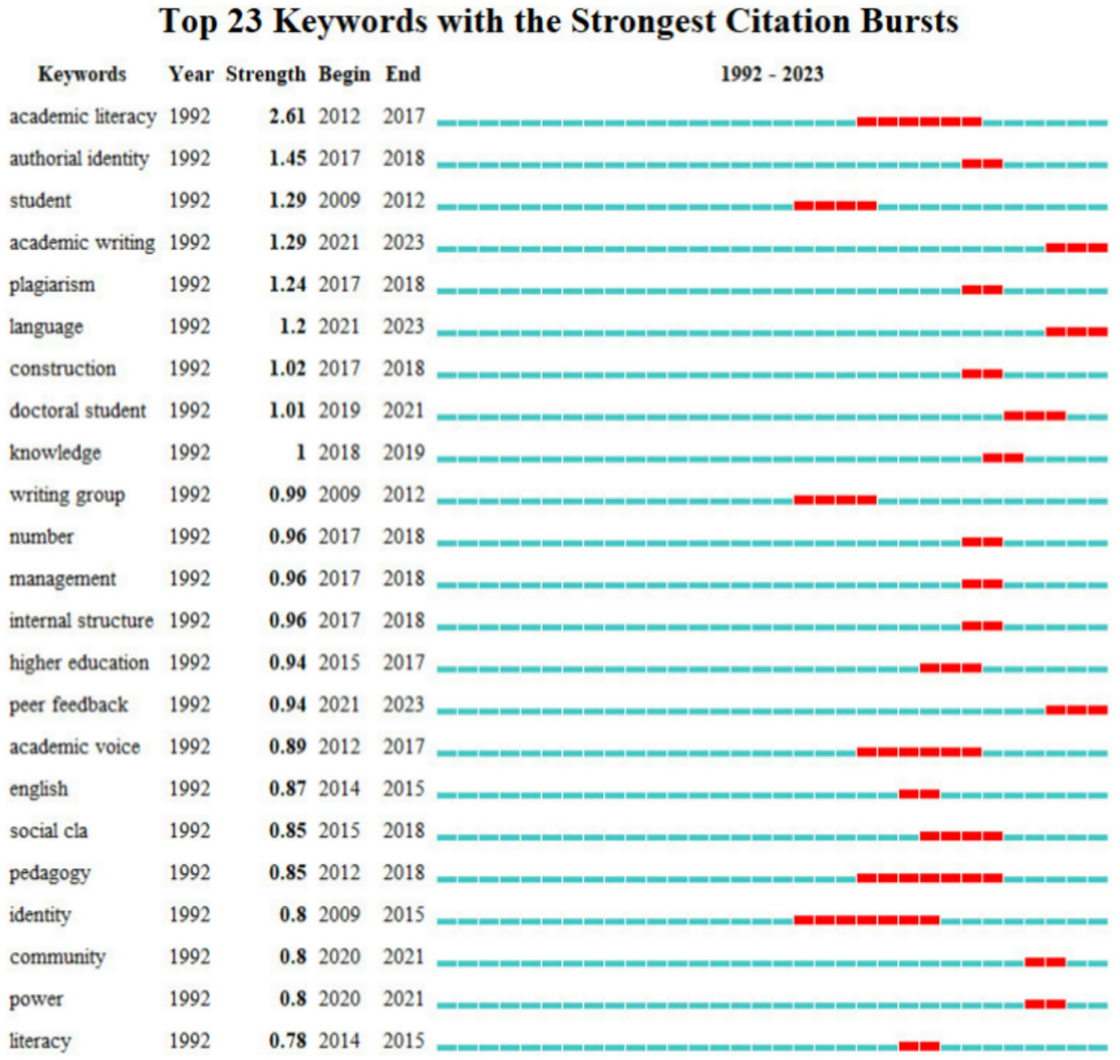
Keywords burst detection.

*CiteSpace* was employed as the tool to analyze data from WoS. The selection criteria were set by thresholds, adjusted multiple times to (1, 1, 10) (1, 1, 10) (1, 1, 10). Two hundred and seventy two nodes and 1,102 lines were obtained, which yielded a line-to-node ratio of 1.5 and provided a solid and appropriate basis for analysis. These nodes and lines were depicted in Figure 3 and visualized over a timeline in Figure 4. The larger font of the node and character in Figure 3 indicated a higher frequency of keywords. A keyword burst detection analysis was conducted, and the results are presented in Figure 5, illustrating a surge in keyword frequency. Table 1 displays all keywords with a frequency of three or more and the initial year of their appearance, excluding search terms.

**Table 1:** Keywords with a frequency of 3 or more.

Keyword	Frequency	The initial year	Keyword	Frequency	The initial year
Academic literacy	12	2012	Plagiarism	4	2017
Student	8	2009	Academic identity	3	2011
Literacy	7	2014	Teacher	3	2011
English	6	2014	Doctoral student	3	2019
Construction	5	2009	Authorial identity	3	2017
Discourse	5	2012	Language	3	2021
Pedagogy	5	2012	Multiliteracy	3	2012
University	5	2017	Doctoral education	3	2009
Writing group	4	2009	Gender	3	2009

#### 3.2.1 Studies focusing on academic writing

It can be concluded from Table 1 that in terms of academic writing, studies are primarily involved in the subfield of literacy concerning high-frequency keywords such as “academic literacy,” “literacy,” and “multiliteracy.” They are closely related to the increasingly diverse population in higher education. According to Figure 5, both “academic literacy” and “literacy” show burst strength, with “academic literacy” exhibiting significantly higher strength than the other keywords and lasting for an extended period. This underscores its importance in the development of this field. Although “multiliteracy” shows no burst strength in Figure 5, it appears relatively early in Figure 4, demonstrating its fundamental role in this field.

In academic writing, literacy is the skillful use of language to understand and communicate a writer’s viewpoints effectively. As a perspective of academic writing, academic literacy (Lea and Street, 2006) is proposed as an alternative to the deficit model and the academic socialization model in response to more flexible and accessible higher education institutions with increasingly diverse student populations. This approach moves beyond traditional views of writing as a set of discrete technical skills in need of fixing, such as grammar or spelling, and instead considers writing as a social practice that varies according to context, culture, and genre. It is related to social identity, power, authority, and meaning-making (Lea and Street, 2006), which emphasizes that students’ academic writing is deeply connected to their understanding of knowledge and the development of their identities. Besides, academic writing is viewed as dynamic and contested, with diverse interpretations of “good writing” across contexts, and critical awareness of the hidden assumptions and values underlying academic practices is encouraged. Academic literacy theory (Lea and Street, 2006) provides a comprehensive framework for understanding the complexities of academic writing in higher education.

Multiliteracy acknowledges the diverse ways people communicate and make meaning in the increasingly globalized world, emphasizing the importance of understanding and valuing diverse languages, dialects, and cultural practices in communication. Multilingual and translingual writers are significant subjects in this field (Canagarajah, 2020). They are typically reflective, knowledgeable, and skilled individuals with transnational or transethnic experiences, adept at navigating the diverse language and literacy environments they encounter in their daily university lives. However, they often face challenges in adapting their multilingual and multiliteracy skills to fit the academic context, occasionally highlighting or downplaying these abilities as needed. Canagarajah (2015) examined how a dialogical pedagogy in multilingual writing classrooms helped students construct their academic voices through a case study. His study further demonstrated that academic writing voice was a hybrid construct that involved negotiating personal identity, cultural background, and academic conventions.

#### 3.2.2 Studies focusing on higher education

Sub-topics focusing on higher education are the second largest part of the literature according to high-frequency keywords in Table 1, including “doctoral student,” “doctoral education,” “university,” “pedagogy,” and “student.” Based on Figures 4, 5, keywords in this field emerged early, accompanied by various bursts of keyword activity throughout its development, such as “writing group” and “peer feedback” in recent years, implying that research in this general field is relatively vibrant.

Doctoral education in universities is the primary research focus, although some studies examine undergraduates as research subjects. Academic literacy provides a frame for designing curriculum and instruction, emphasizing pedagogy in universities (Lea and Street, 2006). Exploring effective pedagogical approaches to improving academic writing is another crucial area of interest within this field. Social support mechanisms are particularly significant in the academic literacy model because they directly address the challenges of navigating writing as a social practice. For instance, writing centers, as a form of writing support, are among the most significant resources. An academic writing center can serve as a mentoring environment and a collaborative learning space, helping students and young academics explore their academic identities and facilitating their transition and transformation (Archer and Parker, 2016).

#### 3.2.3 Studies focusing on identity

Other high-frequency keywords in Table 1 are related to identity concerning “construction,” “academic identity,” “authorial identity,” “plagiarism,” and “gender.” Figure 5 shows that most related burst keywords in this field appeared but lasted only briefly, except for “academic voice.” No burst keywords have emerged recently, indicating research in this field generally lacks vitality.

As Bruce (2008) indicated, effective academic writing required appropriate positioning within the academic community and the ability to display an identity as a scholar. It can be achieved through the construction of an authorial identity. On the other hand, individual academics constantly construct and present their identity as professional “selves” through academic writing. French (2020) pointed out that constructing and maintaining a positive academic (professional) identity were partly, yet significantly, achieved through professional writing in higher education habitus. Various social-cultural identities could influence academic writing (Belcher, 2009), and gender is the most discussed.

Keywords “academic identity,” “authorial identity,” and “plagiarism” are closely related to each other. A robust academic and well-established authorial identity is likely to foster a strong commitment to academic integrity and originality. Improving students’ authorial and academic identity contributes to reducing unintentional plagiarism as they can understand the role of the author better and take a more authorial role in their academic writing.

### 3.3 Visualization of clusters

The cosine index can represent the co-occurrence intensity: the larger the cosine value, the greater the co-occurrence intensity between keywords. This study applied the clustering principles proposed by Callon et al. (1991) to divide the clusters. With the aid of the *CiteSpace* software, a 272*272 keyword matrix was generated. The pair of keywords with the highest cosine index were identified as the theme words in the first cluster. The 272 keywords in the matrix were then sorted in descending order based on their cosine index with either of the keywords. Keywords with non-zero values were selected in descending order, including theme words, and the cluster name was summarized according to the content of the keywords within the cluster. If a cluster contains more than 10 keywords or less than 2 keywords, it will be excluded from being classified as a cluster. After generating a cluster, the keywords within this cluster were removed by deleting the corresponding rows and columns in the matrix, preventing these keywords from being included in subsequent clusters. Those steps were repeated until all keywords with co-occurrence relationships had been clustered (all remaining keywords had a co-occurrence intensity of 0). Forty-one clusters were identified, with 2 excluded because the number of members did not meet the required standards. Finally, 39 valid clusters were obtained through this process.

**Figure 6:**
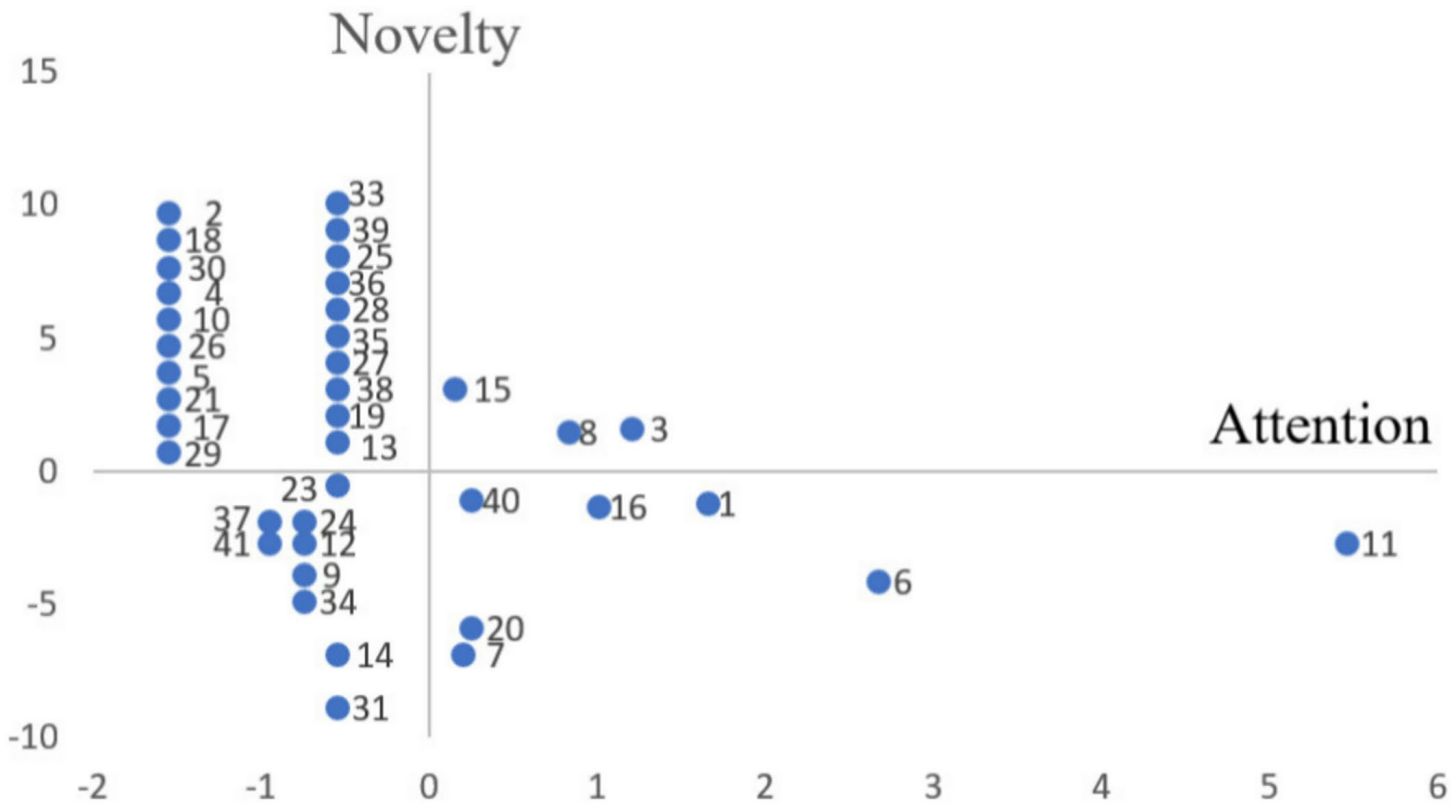
The coordinate diagram of clusters.

The average frequency of keywords within a cluster, minus the average frequency of all keywords, represents the attention level of the cluster. Similarly, the average initial year of appearance for keywords within a cluster, minus the average initial year of appearance for all keywords, represents the novelty level of the cluster. A strategic coordinate diagram was plotted, with attention on the x-axis and novelty on the y-axis, as shown in Figure 6. The names and members of all clusters are listed in Supplementary material.

Clusters in the first quadrant have novelty and attention values greater than 0, suggesting that the research contents represented by these clusters are highly regarded and constitute current hotspots in this field.

Clusters in the second quadrant have a novelty value greater than 0 but an attention value less than 0, indicating that related research contents are novel but have not yet gained the widespread attention they deserve. They are potential future hotspots and will gradually shift to the first quadrant, becoming more established research hotspots as interest increases.

Clusters in the third quadrant have both novelty and attention values less than 0, indicating that their research contents have drawn low attention due to their low novelty, placing them in marginalized research areas. These fields are now somewhat outdated.

Clusters in the fourth quadrant have an attention value greater than 0 but a novelty value less than 0, indicating that the contents represented by these clusters are well-regarded but not the recent research hotspots belonging to foundational research areas.

Through meticulous manual analysis of the collected articles, the clusters were further categorized into four fields: plagiarism related to authorial and academic identity; academic (professional) development related to authorial and academic identity; socio-cultural identity in academic writing; pedagogy of identity construction in academic writing.

In the following discussion section, a comprehensive and in-depth analysis of the clusters and their research contents will address each theme’s specific state of research, its characteristics, and practical implications, particularly regarding plagiarism prevention, pedagogy, and scholarly development. Greater attention will be given to the first and second quadrants because they contain clusters with a high level of novelty and represent current hotspots and substantial potential ones for further research.

## 4 Discussion

### 4.1 An overview of development stages, general trend, and cluster distribution

There are three phases of development in this field: gradual growth (2003–2016), accelerated growth (2017–2020), and accelerated decline (2021–2023). From 2003 to 2016, scholars and institutions began to recognize the significance of identity construction and academic writing in higher education, but this significance remained low. Topics during this period emerged early, laying the foundation for future research in this field. These studies are predominantly positioned in the fourth quadrant. Although these clusters have relatively low novelty, they still receive a certain level of attention due to their foundational importance. The seven clusters in the fourth quadrant are *subjectivity*, *transition experience of novice lecturer*, *development and adaptation in the new era of higher education*, *excellence and performance in academic identity*, *collaborative participation*, *a pedagogy of graduate writing*, and *writing support* (e.g., Belcher, 2009; Gourlay, 2011; James, 2012).

Since 2017, the literature volume increased sharply, reaching its peak in 2020. With the development of higher education, the “publish or perish” culture has intensified (Nygaard, 2017), leading to unprecedented attention on academic writing and identity construction. As a result, a substantial body of literature has rapidly emerged and expanded in this area. This trend aligns with the large number of clusters in the third quadrant, which is the second largest group with nine clusters, although they now appear somewhat outdated. They are *academic voice*, *transitioning from professional work and Master’s coursework to the research dissertation*, *decolonization*, *socio-symbolic function of academic language*, *resistance by L2 writers*, *writing center from the consultant’s perspective rather than students*, *multi-disciplining writing groups*, *engagement*, and *an academic literacies framework investigating research productivity* (e.g., Mitchell, 2017; Okuda and Anderson, 2018; Shaw and Le Roux, 2017).

After 2020, it showed a sharp decline. This finding corresponds to the number of clusters in the first quadrant, which is the fewest, with only three, indicating that there are few current research hotspots in this field and that overall research activity is presently low. Previous research hotspots (in the third quadrant) have become outdated, while new emerging hotspots (in the second quadrant) remain in a latent phase and have yet to materialize fully. As a result, the volume of literature during this period sharply declined. However, it is not at the bottom, consistent with the large number of potential clusters in the second quadrant. Future research on identity may eventually return to a more stable developmental status as emerging hotspots transfer into current ones.

The majority of clusters are distributed in the second quadrant, with 20 clusters accounting for approximately 49% of the total. They currently represent potential future hotspots and related topics that need further exploration. The next two sections will elaborate on the current hotspots in the first quadrant and potential ones in the second quadrant, which require particular attention.

### 4.2 Current hotspots

Three clusters are identified as current hotspots: one related to the field of plagiarism and the other two to sociocultural identity. First, plagiarism is an ethical violation involving using another’s work or ideas without proper attribution, which undermines authorial and academic identity by violating academic integrity norms. Constructing a strong authorial and academic identity can, in turn, help prevent plagiarism. The current hotspot related to plagiarism is *academic integrity*, which has been studied from the perspective of Academic Integrity Socialization. Merkel (2022) found that most students had a limited and traditional understanding of plagiarism, viewing it solely as a violation. They were more focused on cultivating a moral academic identity as writers than on adhering to the academic community’s formal guidelines and norms related to plagiarism. The perspective of Academic Integrity Socialization can address this problem to a certain degree. According to this perspective, students should be provided with a safe and supportive space to explore the *academic integrity* expectations of their institution in a comprehensive and learner-centered manner. Khoo and Kang (2022) investigated undergraduates’ responses to Academic Integrity Socialization. They concluded that when students saw *academic integrity* as integral to their identity as junior scholars, they engaged more meaningfully in scholarly conversations in these spaces and contributed to the academic community with integrity and respect.

Second, the other two current hotspots are related to socio-cultural identity in academic writing. They are *academic literacy* and *English-as-a-second-language discourse*. A*cademic literacy* is novel and popular with the highest burst strength, allowing scholars to examine issues of voice and writer identity in academic writing, which become more complex in multilingual contexts (Robinson-Pant and Wolf, 2017, p. 11). The geopolitics of academic publishing is one of the topics in this field. Through the lens of *academic literacy*, it was found that academics outside the Global North valued the opportunity to publish internationally to gain a voice in the global academic community. International journals are perceived to reach a larger audience, provide more rigorous peer review, and operate more efficiently than local journals (Getahun et al., 2021). *Academic literacy* also provides a theoretical foundation for creating socio-cultural spaces for writing support in alignment with the spatial nature of academic writing. Writing groups, increasingly popular in universities, offer students a space to develop their voice and identity as academic writers (Aitchison and Lee, 2006; Larcombe et al., 2007). This conclusion was confirmed further by Wilmot and McKenna (2018). In addition to writing support forms like writing groups, researchers also identified other ways to enhance academic writing from the perspective of *academic literacy*, such as the appropriate use of various writing genres (Roald et al., 2021). In terms of the scope of application, the academic literacy model should be integrated into the practices of all students, regardless of their linguistic identities (Hathaway, 2015).

Another current hotspot is *English-as-a-second-language discourse*. It explores how identities and voices are formed in the increasingly prevalent transnational environments of today’s globalized society, focusing on English academic discourse in higher education, particularly as a second language in plurilingual or multilingual writing contexts (Langum and Sullivan, 2020). This section presents the topics currently attracting the most attention from scholars, while the next section will elaborate on potential hotspots.

### 4.3 Potential hotspots

Twenty potential hotspots are distributed across the four fields of plagiarism, academic (professional) development, socio-cultural identity, and the pedagogy of identity construction. First, the potential future hotspots related to plagiarism focus on evaluating identity, including *measures of identity* and *discoursal identity*. Pittam et al.’s (2009) six-factor model (SAQ) and Ballantine et al.’ (2013) three-factor model (alternative SAQ) are pioneering *measures* of evaluation. Based on them, scholars continued to refine the measures, illustrate the discursive embodiment of the measures, and elaborate attributes related to authorial identity (Cheung et al., 2017; Cheung et al., 2018; Kanwal et al., 2021). Some scholars have examined these measures in the context of non-English speakers, particularly Chinese students (Ballantine et al., 2018), because English academic writing is becoming increasingly important for multilingual students with the globalization of higher education and the academic community. Preventing plagiarism by enhancing authorial and academic identities is a current hotspot, but its effectiveness depends on solid and reliable evaluation measures (Cheung et al., 2017). Therefore, these measures appear as potential hotspots.

Second, five potential hotspots were identified within the academic (professional) development field. On one hand, they focus on *the spatial nature* of academic writing practice (Beighton, 2020), which aligns with the current hotspot *academic literacy*. Both confirm the importance of a safe and supportive space for promoting academic writing and identity, as students often face alienation and fear accusations of plagiarism due to limited English proficiency, which can negatively impact their relationships with peers and instructors, as well as their sense of belonging in the academic community. Creating such a space is crucial for addressing this issue. This *spatial nature* can be embodied in various forms, such as writing groups and retreats. Writing groups, as spaces for academic writing development, offered a transformative framework that supported proactive student learning through peer interaction (Wilmot and McKenna, 2018). Writing retreats could alleviate the isolation associated with academic writing, thereby improving scholars’ sense of belonging within the academic community and leading to better hedonic and eudaimonic *well-being* for writers when the interventions were sustained (Eardley et al., 2021).

On the other hand, they focus on *the spatial dynamics and transition of academic identity*, particularly among returnees to China from abroad, doctoral students, and educators, because these are the three most representative groups to undergo a spatial transition in the academic community of higher education. The journey to becoming a mature academic is ongoing, as it requires adapting to evolving writing standards and the changing policies of institutions across different regions as needed. The norms and values of academic writing are acquired, negotiated, and sometimes resisted during the transition of doctoral identity to academics (Katila et al., 2019). Doctoral graduates who study abroad and return to work in Chinese universities face significant challenges in *(re)constructing academic identity as returnees*, particularly when adapting to various academic assessment policies in Chinese higher education (Ai, 2019). For educators, academic identity can be developed through the repeated transitions between researcher and teacher roles when engaging in systematic study or teaching and learning practices. The transition is also beneficial for writing *SoTL* (*scholarship of teaching and learning*) *identity* articles for publication, which can lead to greater recognition within the academic community (Healey et al., 2019).

Third, the nine potential hotspots related to sociocultural identity can further be divided into two themes: linguistic factors resulting from the globalization of the academic community and other social factors such as individual experiences, nationality, gender, and ethnicity. The influence of *bilingualism/multilingualism* in constructing multiple identities as an academic writer has been confirmed (Asadolahi and Nushi, 2021). *Translanguaging* originated in bilingual education and was an adaptable strategy to utilize the entire linguistic repertoire during the learning process. It has been proven effective for promoting identity construction when introduced in English for Academic Purposes (EAP) courses (Hiller, 2021). However, the strategy of *translanguaging* should be employed judiciously, as multilingual students perceive *language mixing* in formal writing as inappropriate and potentially undermining their academic identity (Kafle, 2020). Therefore, they tend to restrict their *multilingual* and *multiliterate* skills to conform to institutional standards for conventional academic writing in formal English academic contexts while negotiating their multiple identities in practice (Marshall et al., 2012). In response to this finding, Gagne et al. (2023) suggested that educators could create more positive and inclusive academic environments by recognizing and utilizing students’ diverse linguistic abilities rather than viewing them as challenges to be overcome, to make them effectively use their *multilingual* and *multiliterate* skills. In addition to academic environments, multilingual proficiency and sociocultural identities can influence students’ academic writing engagement. Students with a more positive sociocultural identity and mastery-oriented learning beliefs are more actively involved and can progress more (Zhang and Xu, 2022).

Some other individual and social factors can potentially become hotspots in the future. *Life experience* is one of them because the depth of personal experiences shapes various roles involved in academic identity, such as creator, interpreter, communicator, and presenter (Lo et al., 2020). As a result, personal experience also influences academic writing by shaping the writers’ *self-perception* (Clark and Ivanic, 2013). It supports the finding that writing projects are most meaningful when students have opportunities to connect their writing to personal factors, including the sense of authorship and vision for future writing or identities (Eodice et al., 2019). This explains why *shifting the focus from the final product to the writing process* is a prevalent trend in academic writing pedagogy, as it better leverages students’ experiences by encouraging and sustaining students’ agency and identity in higher education. In addition, gaining a deeper understanding of *gender*, *social class*, *nationality*, and *ethnicity* identities further helps students navigate their approach to academic writing more effectively (Preece, 2018; Danvers et al., 2019; Zhang and Xu, 2022; Pham and Hayden, 2019).

Fourth, there are four potential future hotspots in identity construction pedagogy. Three focus on *doctoral students* and education, further corroborating the findings from the keyword co-occurrence analysis. The function of *mindfulness practices* in doctoral academic writing has been confirmed because they can help participants better understand the writing process and writer identity, accepting both themselves and others as writers through self-reflection, creativity, and joy in writing (Woloshyn et al., 2022). This conclusion is supported by the process of *writing a doctoral thesis*, which includes key elements such as selecting a topic/title for the project, writing the abstract, conducting the literature review, and performing the analysis. Throughout this process, interactions with support networks play a crucial role in shaping, developing, and refining academic identity (Nartey, 2021). Besides, it is proposed that *doctoral student performance* should be evaluated based on the dimension of academic writing (Ward and Brennan, 2020). Specifically, Ward and Brennan (2020) added subdimensions of student-learning identity fit and student-(academic) writing fit to extend Baker and Pifer’s (2015) multidimensional framework of student-doctoral fit. The new model also serves as a tool to develop instruments, such as surveys or assessments, to test specific hypotheses or propositions about *doctoral student performance*.

This study identifies three dimensions of identity involved in academic writing, facilitating a more comprehensive theoretical understanding of how identity is reflected, negotiated, and constructed in academic writing contexts. By employing the strategic coordinate diagram, it visualizes both the current research hotspots and underexplored areas in this field, enabling researchers to understand the current state of research and identify gaps and opportunities for further inquiry, therefore advancing the theoretical landscape.

## 5 Conclusion

### 5.1 Major findings

The literature in this field typically showed a growing trend before 2020 but declined steeply after 2020. The years 2016 and 2020 marked significant milestones in its development. Published articles exhibited a modest increase despite some fluctuations before 2016. Since 2017, it has risen sharply and reached its peak in 2020. After that, it saw a steep decline up until 2022.

Only three clusters can be regarded as current research hotspots. They are distributed in plagiarism and socio-cultural identity and focus on academic integrity, academic literacy, and English-as-a-second-language discourse. The majority of clusters, 20 in total, are identified as potential future hotspots. They are distributed across all four fields: plagiarism, academic (professional) development, socio-cultural identity, and the pedagogy of identity construction.

Potential future hotspots in plagiarism focus on measures of authorial and academic identity, while those in the academic (professional) development field highlight the spatial aspects of academic writing and explore ways to promote academic growth and well-being for various groups in higher education, including doctoral students, instructors, and returnees. The potential future hotspots in the field of socio-cultural identity center on how linguistic and social identities influence academic writing in the context of the globalized academic community. Linguistic factors involve *translanguaging*, *language mixing*, *multiliteracy*, *English language privilege*, and *bilingualism/multilingualism*. Other social-cultural factors include *individual experiences*, *nationality*, *gender*, and *ethnicity*.

Potential future hotspots in the pedagogy of identity construction highlight practical approaches to enhancing identity development and academic writing. Universities are suggested to support *mindfulness practices*, recognize academic identity in writing a *doctoral thesis*, highlight the *process of writing* rather than the final product, and consider academic writing, grounded in a strong sense of identity, as a key *assessment of doctoral student performance*.

Most of the research in this field has been conducted using thematic analysis as a qualitative method. Most clusters are distributed in the second quadrant, indicating a generally high level of novelty, though they have not received the attention they deserve. The second-largest group of clusters is located in the third quadrant, reflecting somewhat outdated topics. Therefore, most studies in this field either exhibit high novelty but lack attention as future hotspots or are overlooked due to being outdated. The current hotspots are relatively rare.

Studies focusing on socio-cultural identity and pedagogy of identity construction are more thriving than the other two, with more clusters representing. However, clusters in the second quadrant related to academic (professional) development account for the most significant proportion, indicating their great potential to generally become hotspots in the future.

### 5.2 Implications

Enhancing authorial and academic identity is an effective way to prevent plagiarism. Students often view plagiarism solely as an ethical violation and fail to understand it within the broader framework of academic writing norms. Providing a safe and supportive space is beneficial for them to examine expectations for academic integrity from a comprehensive, detailed, and learner-centered perspective. Studies on effective and valid measures for evaluating authorial and academic identity offer valuable insights into its development in academic writing.

Based on the spatial nature of academic writing practice, writing supports, such as writing groups, centers, retreats, and mindfulness practice, effectively alleviate isolation and enhance scholars’ sense of belonging within their academic identity, thereby promoting their development as academics.

In the context of socio-cultural identity in academic writing, writing groups can be organized from the perspective of academic literacy, with the academic literacy model integrated into the practices of all students, regardless of their linguistic backgrounds.

Educators should recognize and value students’ multilingual abilities to develop inclusive teaching methods that better support non-native students in English-dominant academic environments. Additionally, the construction of academic identity should be incorporated into doctoral theses, with the focus shifting from the final academic product to the writing process. Authorial identity should be treated as a form of tacit knowledge to be developed, evolving through maturing and gaining experience as a writer.

### 5.3 Limitations

The study is far from flawless, and several limitations must be acknowledged. First, the *CiteSpace analysis* was confined to data derived from the Web of Science (WoS) database due to the data format requirements. While the results are basically representative, they do not capture the full breadth of existing literature. Future research should integrate data from additional databases such as Scopus and ScienceDirect for a more comprehensive analysis.

Second, the names and content of the clusters may have overlapping meanings with less distinct boundaries, as a single publication can address multiple specific themes. Consequently, cluster names primarily reflect the main themes expressed, potentially overlooking the nuanced interconnections between different topics.

Third, the exclusion of studies published in non-English languages inevitably introduces a bias into the analysis of this important topic, thereby reinforcing the dominance of English in research on multilingual writers’ practices. Addressing these limitations in future studies will strengthen the robustness and applicability of the research outcomes.

## Data Availability

The original contributions presented in the study are included in the article/Supplementary material, further inquiries can be directed to the corresponding author.
